# Diabetic ketoacidosis associated with pancreatic involvement in a patient with von Hippel-Lindau syndrome: a case report

**DOI:** 10.3389/fendo.2026.1880422

**Published:** 2026-08-03

**Authors:** Yina Zhang, Futian Liu, Xueqin Cao, Dan Guo, Yuzhu Zhu, Hong Sun

**Affiliations:** 1 Department of Endocrinology and Metabolism, The Fourth Affiliated Hospital of Soochow University, Suzhou Dushu Lake Hospital, Medical Center of Soochow University, Suzhou, Jiangsu, China; 2 Department of Endocrinology and Metabolism, Kunshan Hospital of Chinese Medicine, Suzhou, Jiangsu, China

**Keywords:** diabetic ketoacidosis, gene mutation, pancreatic cysts, specific types of diabetes, von Hippel-Lindau syndrome

## Abstract

**Background:**

Von Hippel–Lindau (VHL) disease is a rare autosomal dominant disorder predisposing individuals to multisystem neoplasms. While pheochromocytoma is a well-recognized endocrine manifestation of VHL, pancreatic involvement leading to clinically significant endocrine dysfunction is less frequently emphasized. We report a case of VHL syndrome in which diabetic ketoacidosis (DKA) occurred in the context of underlying pancreatic pathology, highlighting a potential but underexplored metabolic complication in these patients.

**Case summary:**

A 31-year-old man came to the emergency department with a three-year history of polydipsia, polyuria, and hyperglycemia, and was diagnosed with diabetic ketoacidosis (DKA). His history included resection of cerebellar hemangioblastoma in 2009 and again in 2017, as well as subtotal pancreatectomy (head and body) for pancreatic lesions in 2017. At that time, his blood glucose was normal. His mother and maternal aunt both had hemangioblastoma. During this admission, we found severely impaired islet function in the residual pancreatic tail. Autoimmune diabetes was ruled out by negative anti-GAD, anti-IA-2, and anti-ZnT8 antibodies. There was no sign of infection or steroid use. Genetic testing showed a heterozygous deletion in exon 3 of the VHL gene, confirming VHL syndrome. With no other triggers identified, we attributed the DKA to acute decompensation from markedly reduced beta-cell reserve, likely due to both prior pancreatic resection and chronic VHL-related pancreatic disease. However, without serial imaging or histopathology to confirm progressive changes in the remnant tail, we could not establish a definitive causal relationship.

**Conclusion:**

This case highlights that DKA can occur as an acute metabolic decompensation in patients with VHL-related pancreatic disease, particularly in those with prior pancreatic resections and progressive beta-cell loss over time. Nevertheless, the exact pathophysiological link between VHL-associated pancreatic involvement and the onset of DKA remains speculative and likely multifactorial. Clinicians should maintain vigilance for endocrine pancreatic insufficiency in VHL patients who present with unexplained hyperglycemia, even in the absence of a prior diabetes diagnosis. Further studies and accumulated case evidence are needed to clarify the mechanisms and risk factors for DKA in this specific population.

## 1 Introduction

Von Hippel-Lindau syndrome (VHL) is an inherited disorder caused by mutations in the *VHL* gene located on chromosome 3. Affected individuals are predisposed to a spectrum of multisystem neoplasms, including central nervous system hemangioblastomas (CHB), retinal hemangioblastomas (RHB), renal cell carcinoma or cysts, pancreatic tumors or cysts, pheochromocytomas, endolymphatic sac tumors, and epididymal or broad ligament cysts (1, 2). Epidemiological data for VHL syndrome in China remain unavailable; however, prevalence estimates in European and American populations range from 1 in 38, 000 to 1 in 91, 000 (3). In 1993, Latif et al. (4) identified the VHL gene through positional cloning and mapped it to chromosome 3p25–26. The gene encodes the VHL protein (pVHL), which assembles into an E3 ubiquitin ligase complex—along with elongin B, elongin C, and other components—that targets hypoxia-inducible factor alpha (HIF-α) subunits for proteasomal degradation. Pathogenic VHL mutations, including missense, nonsense, small or large deletions, and splice-site alterations, disrupt this degradative function, leading to intracellular accumulation of HIF-α. Accumulated HIF-α activates the transcription of downstream oncogenic pathways, including vascular endothelial growth factor (VEGF) and platelet-derived growth factor (PDGF). Subsequent overexpression of these factors promotes angiogenesis, cell proliferation, and migration, ultimately driving tumorigenesis in multiple organ systems.

Here, we report a case of VHL syndrome due to a heterozygous deletion in exon 3 of the VHL gene, diagnosed at the Fourth Affiliated Hospital of Soochow University.

## 2 Case presentation

A 31-year-old man presented to the emergency department with a three-year history of polydipsia and polyuria, unintentional weight loss of 5 kg over the preceding six months, and a random capillary glucose reading of 18 mmol/L. Initial laboratory evaluation revealed an HbA1c of 12.9%, venous glucose of 18.8 mmol/L, ketonuria, and an arterial pH of 7.332. Serum bicarbonate was 19.1 mmol/L (normal 22–29 mmol/L). Serum potassium was 3.39 mmol/L, sodium 135.9 mmol/L, calcium 2.02 mmol/L, phosphorus 0.52 mmol/L (normal 0.85–1.51 mmol/L), and chloride 101 mmol/L (normal 98–106 mmol/L). The anion gap was elevated at approximately 15.8 mmol/L (normal range 8–12 mmol/L). Serum ketone level was 4.9 mmol/L (normal < 0.6 mmol/L), confirming significant ketosis. Based on the arterial pH of 7.332 and bicarbonate of 19.1 mmol/L, this episode was classified as mild diabetic ketoacidosis (DKA) according to current guidelines. The combination of hyperglycemia, metabolic acidosis with an elevated anion gap, and elevated serum ketones confirmed the diagnosis of DKA.

His surgical history was notable for two prior resections of cerebellar hemangioblastoma (2009 and 2017). During the March 2017 operation, intraoperative findings revealed multiple cystic lesions throughout the pancreas. In the pancreatic head, several lesions measuring approximately 1×1×1 cm each were identified. In the pancreatic body, a larger cystic lesion measuring approximately 8×5×5 cm was found, with a firm solid nodule on its capsule and adhesions to surrounding tissues. In the pancreatic tail, a cystic lesion measuring approximately 3×3×2 cm was also noted. The surgical team performed a subtotal pancreatectomy with portal vein repair, splenic vein repair, and cholangioplasty under general anesthesia, resecting the head and body while intentionally preserving the pancreatic tail. This decision was based on the surgeon’s judgment that maintaining some residual islet mass was necessary to avoid immediate and severe postoperative endocrine insufficiency, as the tail lesion appeared similar in gross morphology to those in the resected specimens and was not considered life-threatening at that time. No glucose abnormalities were documented perioperatively or during follow-up prior to the current admission. Notably, detailed surgical records or histopathological confirmation of the tail lesion were not available for further verification, and no postoperative imaging follow-up was performed to monitor for changes in the remnant tail until the current admission. Family history was additionally significant for brain tumors in his mother and maternal aunt, with his mother also having a history of renal cell carcinoma.

On physical examination, the patient’s vital signs were as follows: temperature 36.3 °C, pulse 110 beats per minute, respirations 18 breaths per minute, and blood pressure 102/74 mmHg. His height was 178 cm and weight 50 kg, with a body mass index (BMI) of 15.8 kg/m^2^. He appeared emaciated but was alert and oriented, in no acute distress. Old surgical scars were noted on the scalp and abdomen. No other significant abnormalities were observed on physical examination.

Laboratory evaluation was undertaken to investigate the etiology of his insulin-deficient diabetes and multisystem involvement. Islet function was evaluated using a 75g oral glucose tolerance test (OGTT). For three days before the test, the patient maintained a normal diet with at least 150 g of daily carbohydrate intake, and medications that could affect glucose metabolism were withheld. After an overnight fast of at least 8–12 hours, a baseline blood sample was collected. The patient then drank a solution containing 75 g of anhydrous glucose dissolved in 250–300 mL of water within 5 minutes. Blood samples for C-peptide and insulin were collected at 0.5, 1, 2, and 3 hours after glucose loading, with the timing starting from the first sip. The reference range for fasting C-peptide is 0.37–1.47 nmol/L, and for fasting insulin is 2.6–24.9 μU/mL. In this patient, C-peptide levels were profoundly suppressed across all time points (0.06, 0.11, 0.23, 0.40, and 0.53 nmol/L at 0, 0.5, 1, 2, and 3 hours, respectively). Although the C-peptide levels at 0.5 and 1 hour increased to approximately 5–6 times the fasting level, and to 4–5 times at 2 hours and 2–3 times at 3 hours, the absolute values remained far below the normal fasting reference range (0.37–1.47 nmol/L), with the peak value of 0.53 nmol/L barely reaching the lower limit of normal. Insulin levels were similarly low (<0.40, 1.44, 2.83, 5.34, and 6.35 μU/mL at the corresponding time points), with the peak value of 6.35 μU/mL remaining far below the normal fasting reference range (2.6–24.9 μU/mL). Moreover, the peak response occurred at 3 hours rather than the expected 0.5–1 hour peak, indicating a delayed and blunted secretory response. These findings confirmed severe β-cell dysfunction and absolute insulin deficiency. Autoimmune diabetes was excluded by negative results for anti-glutamic acid decarboxylase (GAD), anti-insulinoma-associated antigen-2 (IA-2), and anti-zinc transporter 8 (ZnT8) antibodies.

Hormonal evaluation revealed elevated gonadotropins (luteinizing hormone [LH] 19.80 IU/L, follicle-stimulating hormone [FSH] 15.50 IU/L) with normal testosterone and estradiol. Imaging studies were subsequently performed. Scrotal ultrasound revealed reduced volume of the right testis, confirming right testicular hypoplasia. Findings in the left testis were suggestive of sperm stasis. This profile is consistent with compensated hypergonadotropic hypogonadism secondary to congenital unilateral testicular dysgenesis.

Bone metabolism assessment demonstrated severe vitamin D deficiency (25-hydroxyvitamin D 7.9 ng/mL), hypocalcemia (2.02 mmol/L), hypophosphatemia (0.52 mmol/L), and an elevated bone resorption marker (β-crosslaps 1.06 ng/mL). Bone mineral density measurement revealed Z-scores of –3.2 at both the lumbar spine and femoral neck, confirming the diagnosis of severe osteoporosis.

**Figure 1:**
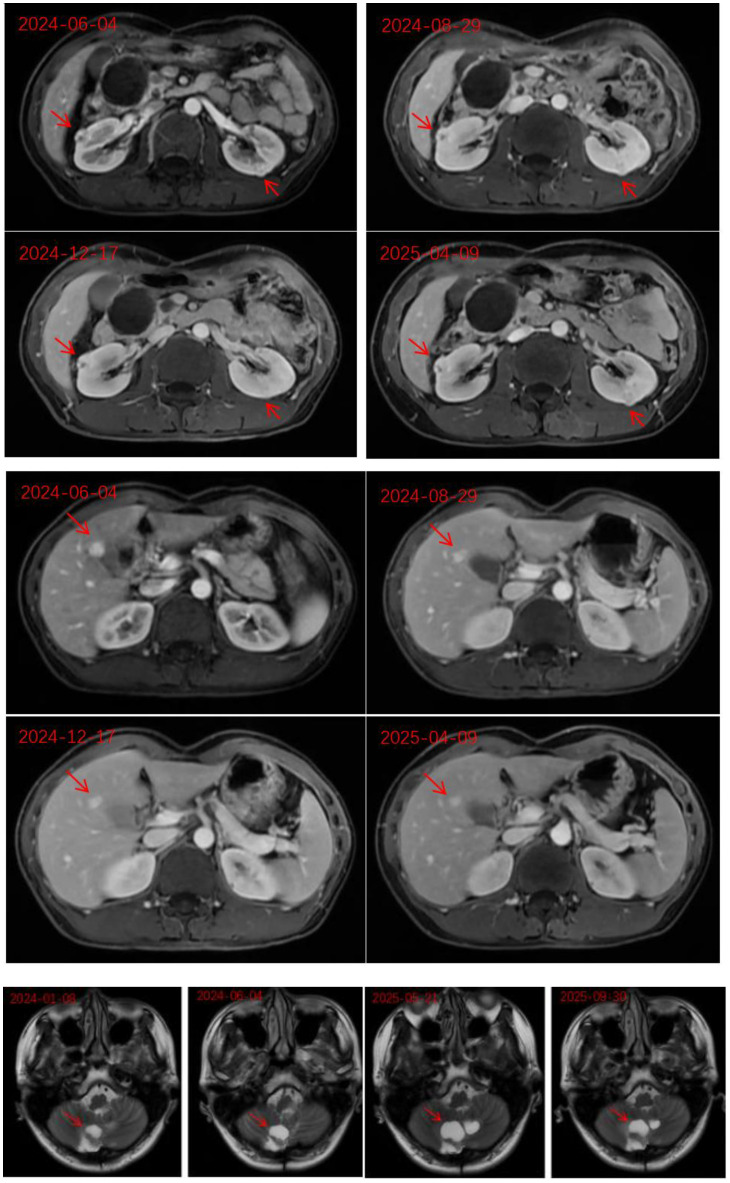
Abdominal and pelvic contrast-enhanced MRI of the abdominal and pelvic cavity indicates two highly enhanced lesions in both kidneys and abnormally enhanced lesion in the left hepatic lobe, with follow-up showing the lesions to be similar to previous assessments; Cranial MRI indicates multiple abnormal signals in the cerebellum, medulla oblongata, and fourth ventricle, with follow-up suggesting progression of the cranial lesions which later reduced in size.

Post-contrast magnetic resonance imaging demonstrated the following findings (**Figure 1**): 1. post-pancreatectomy changes with a residual pancreatic tail *in situ*, which contained a cystic lesion, multiple cystic lesions surround the duodenum. 2. Bilateral renal hyperenhancing lesions and complex renal cysts. 3. Postoperative changes related to a cerebellar hemangioblastoma, with abnormal signal intensities present in both cerebellar hemispheres, the left medulla oblongata, and the fourth ventricle—findings that remained stable compared to prior imaging. 4. Right testicular hypoplasia. 5. A enhancing hepatic lesion. However, no prior abdominal imaging was available for comparison, and serial imaging or histopathological evidence documenting progressive destruction of the residual pancreatic tail was lacking.

**Figure 2:**
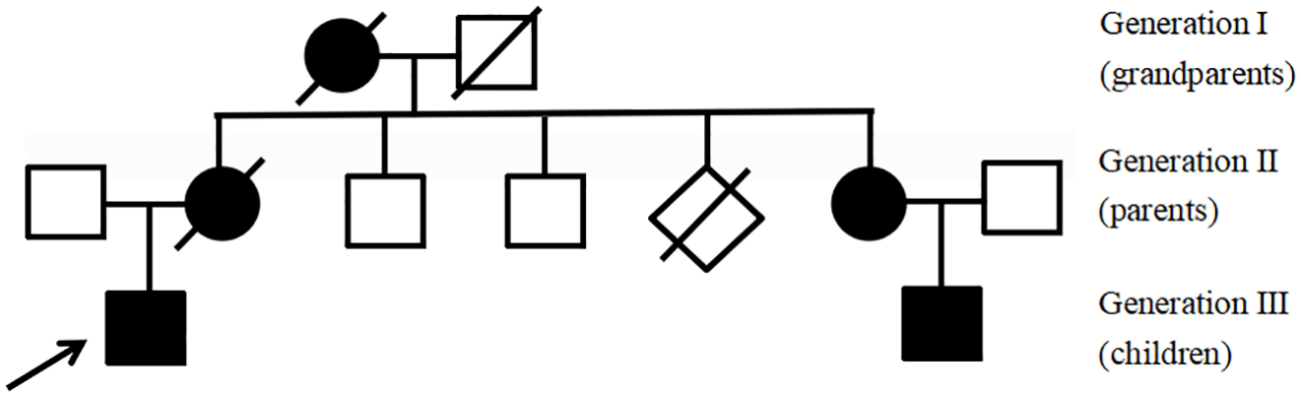
Genetic pedigree chart of the patient.

**Figure 3:**
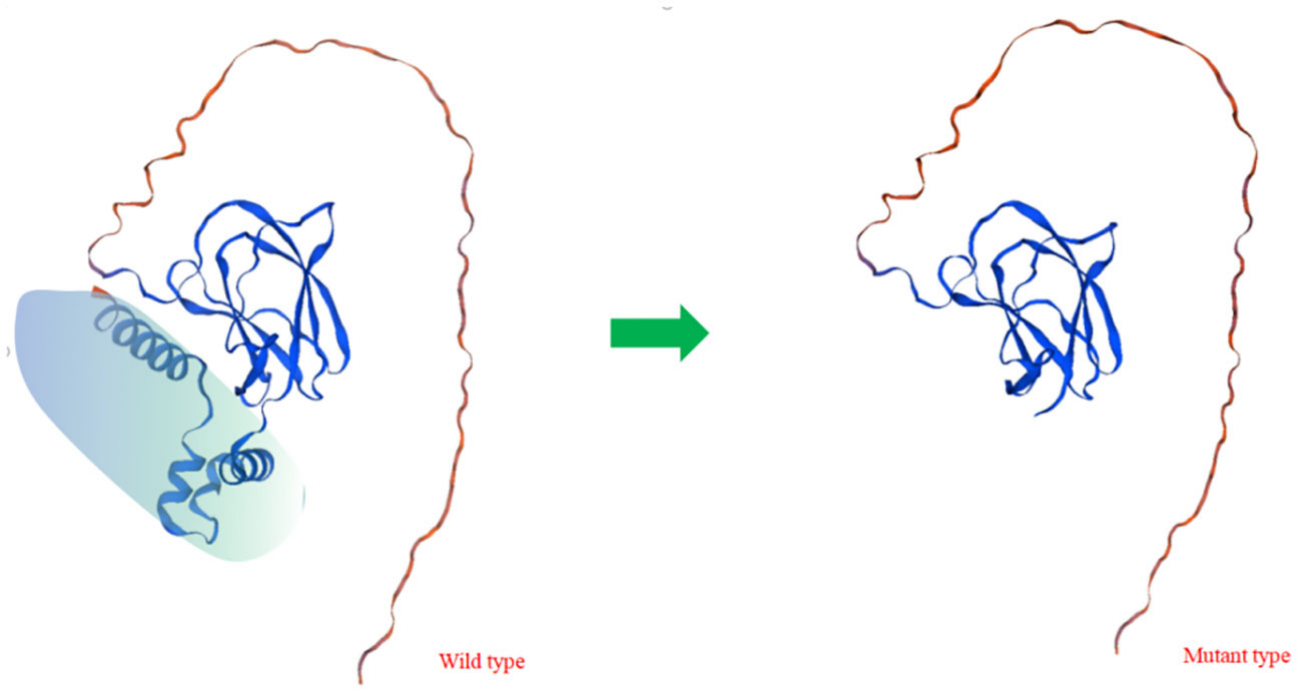
Predicted tertiary structure model diagrams of wild-type and mutant amino acid proteins. The green-labeled parts represent the truncated and lost protein regions after the deletion of Exon 3.

**Figure 4:**
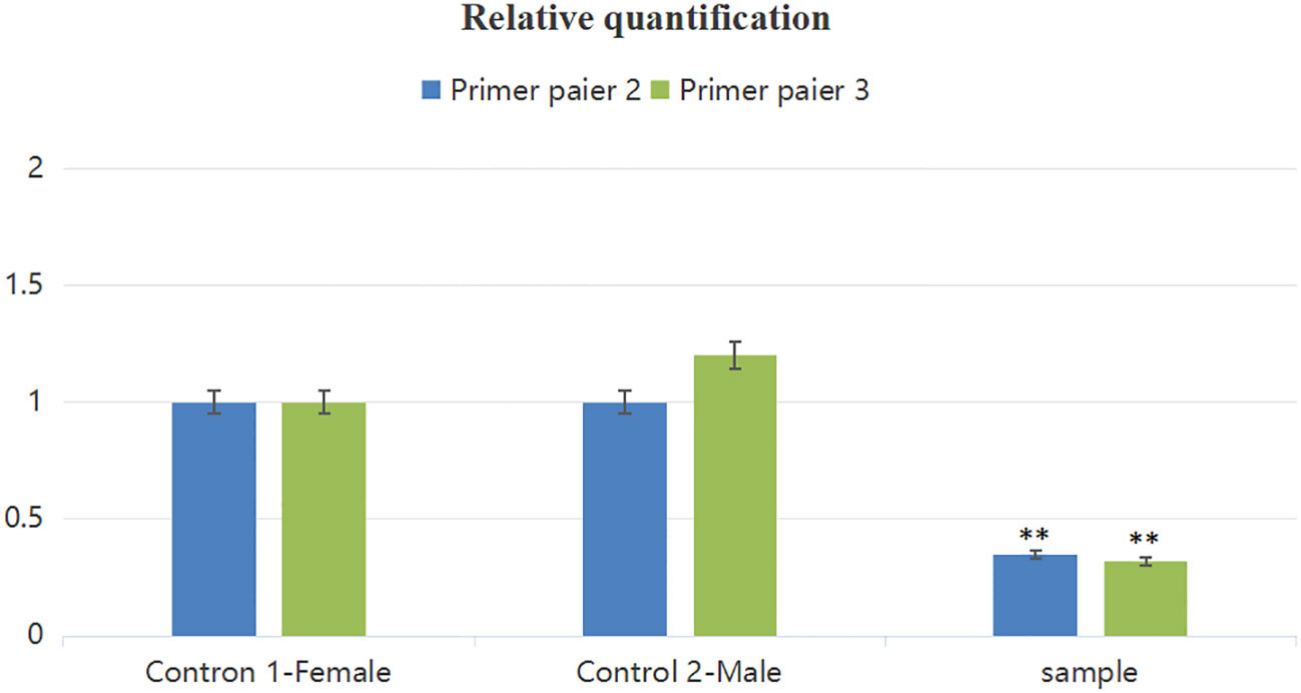
Relative quantification of target gene expression detected by qPCR. Compared with the two control groups, the expression level of the target gene in the sample group was significantly down-regulated, with a highly statistically significant difference (P<0.01) (**P < 0.01).

Based on the constellation of CNS hemangioblastomas, pancreatic cysts, renal lesions, and testicular dysgenesis, along with a strong family history, VHL syndrome was suspected. A pedigree of the patient’s family was constructed (**Figure 2**). This was subsequently confirmed by genetic testing (**Figures 3**, **4**; **Supplementary Figure 1**), which identified a heterozygous deletion at chromosome 3:10188571-10195715 (copy number = 1), involving exon 3 of the VHL gene (NM_000551.3: c.464_642del). This mutation is predicted to result in a truncated protein consisting of 154 amino acids, compared to the wild-type protein of 213 amino acids, thereby establishing the definitive diagnosis of VHL syndrome.

DKA was managed with insulin pump therapy, intravenous fluids, and ketone correction, which successfully controlled his hyperglycemia. Given his absolute insulin deficiency, he was sensitive to exogenous insulin and required close monitoring to avoid hypoglycemia, especially during transition from acute management to maintenance therapy. At discharge, we prescribed a basal-bolus regimen consisting of once-daily insulin glargine for basal coverage and preprandial insulin aspart for meal-time coverage, with clear dose-adjustment instructions based on carbohydrate intake and self-monitored blood glucose levels. We discharged him on a basal-bolus regimen with clear dose-adjustment instructions based on carbohydrate intake and self-monitored glucose. On admission, he was severely underweight (BMI 15.78 kg/m^2^). A dietitian recommended a high-calorie, balanced diet with frequent small meals to increase energy intake while minimizing glycemic spikes. In addition, calcium and vitamin D supplementation was initiated for osteoporosis. For his VHL syndrome, the patient was started on belzutifan (MK-6482) 80 mg daily, a HIF-2α inhibitor that targets the key molecular pathway (5). His overall management also incorporated a multidisciplinary approach, including regular imaging surveillance and genetic counseling.

A cranial MRI in May 2025 demonstrated interval progression since June 2024, with a slight increase in the extent of abnormal signals within the cerebellum, medulla oblongata, and fourth ventricle, as well as new miliary enhancing nodules in both cerebellar hemispheres highly suggestive of metastasis. The patient was subsequently started on belzutifan 80 mg daily. Follow-up imaging in September 2025 showed a favorable response, with a reduction in the extent of the cerebellar, medullary, and fourth ventricular lesions and a decrease in the enhancing nodules.

Due to belzutifan-induced anemia, the dose was reduced to 40 mg daily in October 2025; stable glycemic control was maintained throughout. In July 2025, prior to the dose adjustment, ophthalmologic examination had revealed a new retinal hemangioblastoma in the left eye, which was successfully treated with laser photocoagulation. Laser photocoagulation and cryotherapy are the mainstays of surgical management of RHB (6). Given the small size of the tumor, the patient remained asymptomatic, with no visual impairment or blindness. Annual fundoscopic surveillance was recommended. Renal lesions remained stable on follow-up imaging (**Figure 1**). The patient’s clinical timeline is summarized in **Supplementary Figure 2**. A multidisciplinary approach involving neurosurgery, urology, endocrinology, and genetic counseling was established for long-term surveillance.

## 3 Discussion

### 3.1 Clinical diagnosis and genetic basis of VHL syndrome

The diagnosis of VHL syndrome relies on established clinical criteria and genetic confirmation (7–10).

Major Clinical Manifestations include: 1) CHB (Including RHB); 2) Endolymphatic Sac Tumors (ELSTs) (11); 3) Renal cell carcinomas (RCC); 4) Pheochromocytomas, paragangliomas, and/or glomus tumors; 5) Neuroendocrine tumors and/or multiple pancreatic cysts.

Clinical Diagnosis is confirmed if a patient presents with: (a) at least two CHBs; or (b) one CHB plus one other major manifestation; or (c) one major manifestation plus a positive family history or a known pathogenic VHL mutation (12).

Genetic Diagnosis as Gold Standard: Advances in genetic testing now enable the detection of VHL mutations in nearly 100% of cases. Identification of a pathogenic mutation is sufficient for diagnosis, with genetic testing being particularly crucial for atypical cases or familial screening. In cases of novel mutations, functional studies at the mRNA and protein levels are recommended to confirm pathogenicity (13). Approximately 20% of VHL patients in China have large fragment deletions, and mosaicism can occur, both of which should be considered during genetic testing.

### 3.2 VHL classification

VHL disease is internationally classified into two types based on the presence or absence of pheochromocytoma. Type 1 patients have a high incidence of hemangioblastomas and renal cell carcinomas but no pheochromocytoma. It is further subdivided into type 1A (with RCC) and type 1B (without RCC). Gene mutations in these patients often lead to a complete loss of VHL protein function. Type 2 patients have pheochromocytoma and are divided into three subtypes: 2A (without RCC), 2B (with RCC), and 2C (pheochromocytoma only) (14). Most Type 2 cases are caused by missense mutations in the *VHL* gene, resulting in a single amino acid change.

### 3.3 Advances in targeted therapy

The management of VHL-associated tumors has evolved with the introduction of targeted therapies. Belzutifan, a HIF-2α inhibitor, represents a significant advance in this field. The U.S. FDA approved this drug in 2021. It acts by inhibiting HIF-2α dimerization, thereby blocking the downstream signaling that drives angiogenesis and tumor growth in VHL syndrome (5). Clinical trial data have reported objective response rates of 67% for renal cell carcinoma, 91% for pancreatic neuroendocrine tumors, and 48% for central nervous system hemangioblastomas (15, 16).

In our patient, we started belzutifan at 80 mg daily after detecting multiple progressive intracranial metastatic lesions. Follow-up imaging demonstrated notable shrinkage of these lesions and a decrease in nodule count. This response confirms the drug’s efficacy against central nervous system involvement in VHL disease. However, the patient developed anemia secondary to treatment, a well-documented adverse effect of belzutifan. Clinical trials have reported this complication in 89% of treated patients (17). We accordingly reduced the dose to 40 mg daily. This case illustrates that belzutifan can achieve robust antitumor activity in VHL-related CNS disease, yet its use demands vigilant monitoring for toxicity and individualized dose adjustment to maximize clinical benefit.

### 3.4 Pathophysiological mechanisms in the present case

This case illustrates a cascade of multi-system complications stemming from VHL. In this case, the patient was diagnosed with 1. DKA, 2. VHL, 3. Testicular Dysgenesis, 4. Hypergonadotropic Hypogonadism, and 5. Osteoporosis. Further investigations, including insulin, C-peptide, and insulin-like growth factor tests, revealed impaired islet function. Several factors may have contributed to the development of DKA in this patient, and their respective roles merit careful analysis based on the clinical timeline.

The patient’s prior subtotal pancreatectomy (resection of the pancreatic head and body) represents a well-established risk factor for pancreatogenic (type 3c) diabetes (18). Surgical resection of pancreatic parenchyma directly reduces beta-cell mass. A systematic review and meta-analysis of 82 studies involving 13, 257 patients reported an overall prevalence of new-onset diabetes after partial pancreatectomy of 17.1% (19). In this patient, however, no glucose abnormalities were documented at the time of surgery. This observation is clinically significant. The pancreatic tail, which contains the majority of insulin-secreting β-cells, was intentionally preserved during surgery. This likely provided sufficient islet cell reserve to maintain normal glucose homeostasis in the immediate postoperative period. However, the resection of the head and body—while not immediately diabetogenic—did reduce the total β-cell mass, creating a state of diminished reserve.

Over the following years, the patient developed progressive polydipsia and polyuria beginning in 2021, approximately four years after surgery. This delayed onset of diabetic symptoms suggests that factors beyond the surgical resection itself were responsible for the progressive decline in β-cell function. A study evaluating the association between pancreatic cysts and diabetes mellitus in 36 VHL patients found that patients with a larger area covered by pancreatic cysts were significantly more likely to have diabetes than those without (20). The residual pancreatic tail contained a cystic lesion consistent with VHL-related pancreatic involvement, and we hypothesize that progressive changes in this lesion—rather than the static effect of the prior surgery—contributed to the gradual functional impairment of residual β-cells. This interpretation is supported by the patient’s profoundly suppressed C-peptide levels (0.06–0.53 nmol/L) and the absence of autoimmune diabetes, confirming severe insulin deficiency in the setting of pancreatic disease. The three-year symptomatic period prior to DKA reflects a gradual process of β-cell functional decline over time.

Thus, while the prior subtotal pancreatectomy reduced overall β-cell reserve and may have contributed to the baseline risk, the delayed clinical presentation of diabetes years after surgery suggests that the progressive dysfunction of β-cells was more likely driven by ongoing VHL-related pancreatic pathology in the remnant tail. In a single case report, we cannot precisely quantify the relative contributions of these two factors. However, the temporal sequence—surgery without immediate diabetes, followed by gradual onset of symptoms and eventual DKA—supports the interpretation that VHL-related progression in the residual pancreas played a predominant role in the development of insulin deficiency. The mechanism may involve progressive dysfunction of the residual pancreatic tissue caused by VHL-related cystic lesions. However, we acknowledge that we lack serial imaging or histopathological confirmation of changes in the remnant tail over time, which limits our ability to establish a definitive causal relationship between the progression of the pancreatic lesion and the decline in β-cell function. In addition, fecal elastase-1 was not measured to evaluate pancreatic exocrine function, as this assay is not available at our institution. This test would have provided additional support for the diagnosis of pancreatogenic diabetes, and its absence is a limitation of our case. These limitations prevent us from fully characterizing the underlying pathophysiology in this single case report.The limitation has been explicitly acknowledged, and we emphasize the importance of monitoring for pancreatogenic diabetes in VHL patients who undergo pancreatic resections.

Male patients with VHL disease often present with epididymal cystadenomas, which can affect one or both sides, but typically do not impact fertility (1). Studies using a conditional knockout mouse model for VHL have shown that male VHL-deficient mice have abnormally small testes and a significant reduction in sperm count, ultimately leading to infertility (21). Research has found that male mice carrying VHL mutations exhibit testicular developmental abnormalities and impaired spermatogenesis. Additionally, various proteins interacting with pVHL30 have been identified in human testes, which are involved in spermatogenesis and reproductive metabolism, confirming the critical role of pVHL in normal gonadal development in humans (22). However, there are currently no reports of gonadal dysfunction in VHL patients. Whether this finding is causally linked to VHL syndrome or represents an incidental congenital anomaly is unknown. We consider it more likely to be an incidental finding, although we cannot definitively exclude a contributory role of VHL-related pathways. This patient exhibits a distinct endocrine profile characterized by elevated pituitary gonadotropins (LH, FSH) in combination with normal testosterone levels, resulting from congenital unilateral testicular dysgenesis. The dysfunctional testis, due to insufficient testosterone secretion, fails to provide adequate negative feedback to the pituitary gland. This leads to a compensatory increase in the secretion of high levels of LH and FSH (hypergonadotropism). Driven persistently by the elevated LH, the healthy contralateral testis compensates by enhancing its testosterone production, thereby maintaining peripheral blood testosterone levels within the normal range. Consequently, the condition is fundamentally a hormone imbalance triggered by a primary testicular defect (unilateral dysgenesis). The pituitary function remains normal and responsive, and through the compensatory overwork of the healthy testis, the body successfully achieves critical testosterone homeostasis.

The patient also has severe osteoporosis, with Z-scores of -3.2 at both the lumbar spine and femoral neck. Laboratory evaluation revealed a markedly low 25-hydroxyvitamin D level (7.9 ng/mL; normal ≥20 ng/mL) and an elevated β-crosslaps level (1.06 ng/mL; normal 0.225–0.936 ng/mL), confirming increased bone resorption. Serum parathyroid hormone (PTH) was 35.2 pg/mL (normal 15–65 pg/mL), which is within the normal range. This argues against clinically significant secondary hyperparathyroidism as the primary driver of bone loss.

The patient’s endocrine profile also included elevated gonadotropins (LH, FSH) with a single testosterone measurement within the normal range, indicating a compensated hypergonadotropic state. We acknowledge that a single testosterone measurement may not fully reflect gonadal function. The elevated gonadotropins suggest primary gonadal dysfunction; the normal testosterone level likely represents a compensatory response of the remaining healthy testicular tissue. However, we cannot determine whether testosterone reserve is adequate under physiological stress, as we did not perform human chorionic gonadotropin (hCG) stimulation testing or repeat testosterone measurements. Additionally, inhibin B—a more sensitive marker of Sertoli cell function and spermatogenesis—was not measured, as this assay is not available at our institution. These limitations prevent us from fully characterizing the degree of gonadal dysfunction.

We therefore consider that the patient’s osteoporosis is likely multifactorial. Vitamin D deficiency is an important contributor, but the absence of frank secondary hyperparathyroidism suggests that additional mechanisms are involved. The compensated hypergonadotropic state, with potentially inadequate testosterone reserve, may have contributed to bone loss despite a single normal testosterone value. We cannot definitively determine the relative contributions of these factors in a single case report. Further evaluation—including hCG stimulation testing, repeat testosterone measurements, and inhibin B levels—would be needed to clarify the role of gonadal dysfunction in this patient’s bone disease.

### 3.5 Literature review insights

**Figure 5:**
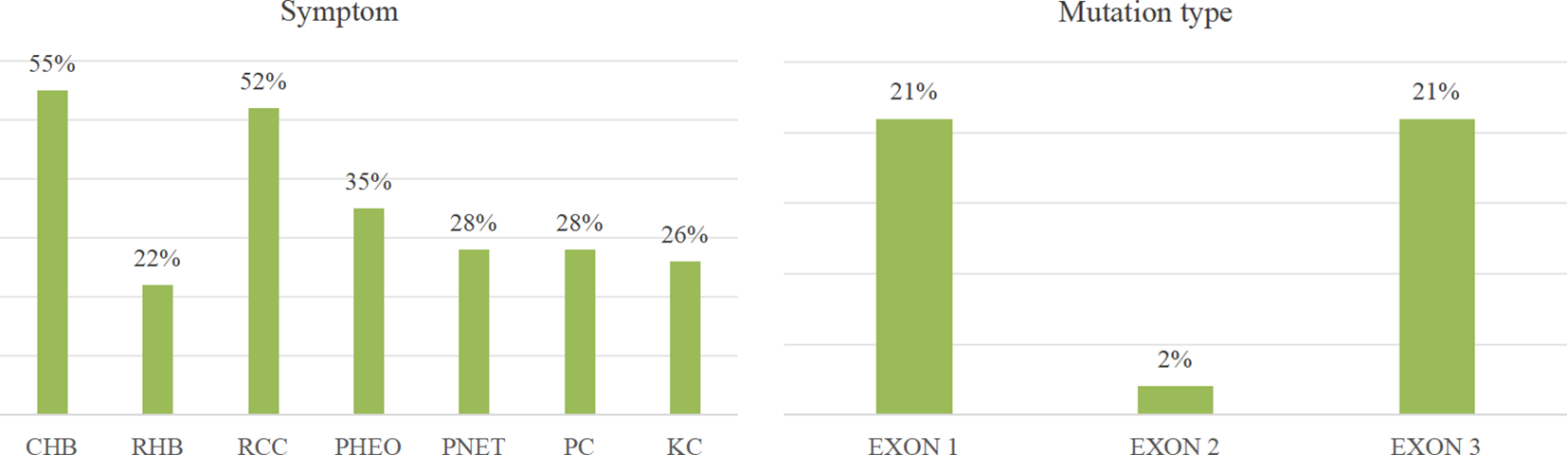
Summary of 58 cases of VHL patients. HB, hemangioma; CHB, CNS hemangioblastoma; RHB, retinal hemangioblastoma; PHEO, pheochromocytoma; PNET, pancreatic neuroendocrine tumor; PC, pancreatic cyst; RCC, renal cell carcinoma; KC, renal cyst.

To better characterize the clinical and genetic features of VHL syndrome, we conducted a focused literature review of VHL-related case reports. We searched PubMed from inception to December 2025 using the following search strategy: (“Von Hippel-Lindau disease” OR “VHL syndrome” OR “VHL”) AND (“case report” OR “case series”). We included original case reports and case series published in English that reported individual-level clinical and genetic data on VHL patients. We excluded reviews, editorials, letters, and studies that did not provide sufficient patient-level information. Two authors independently screened titles and abstracts, and full texts of potentially eligible articles were retrieved and reviewed. Discrepancies were resolved by discussion. From the initial search results, we identified 58 case reports that met our inclusion criteria. A summary of the key characteristics of these 58 cases is presented in **Figure 5**; **Table 1**. Data extracted included year of publication, patient age and sex, VHL gene mutation type, organ systems involved, and reported endocrine or metabolic complications. These cases demonstrated diverse clinical presentations, with the majority reported from China and a male-to-female ratio of nearly 2:1. Most patients presented with CNS hemangioblastoma, renal cell carcinoma, or pheochromocytoma, with incidences of 55%, 52%, and 35%, respectively, and a young median age of onset (35 years). Regarding genetic mutations, exons 1 and 3 were confirmed as major hotspots, with significantly higher mutation rates than other regions. Mutation types included missense, nonsense, and deletion mutations, consistent with the known diversity of the *VHL* mutation spectrum. This analysis aims to provide comprehensive reference materials for clinical practice, supporting the precise diagnosis and effective management of VHL disease.

**Table 1:** Summary of clinical and genetic characteristics in 58 reported cases of VHL syndrome.

ID	Time	Gender	Age	Country	VHLgene mutation	Family medical history	Symptom
P1 (2)	2022	M	20	CHINA	c.208G>A	Yes	RHB
P2 (23)	2022	M	50	Nepal	No	No	RHB, CHB, KC
P3 (24)	2022	F	45	Morocco	No	No	RHB, CHB
P4 (25)	2021	M	12	CHINA	c. 257C>T	Yes	RHB
P5 (26)	2023	F	45	CHINA	No	No	RHB, RCC
P6 (27)	2023	M	38	Italy	c.464-1G>A	No	RHB, RCC
P7 (28)	2023	F	28	Nepal	c.473T>C	No	HB, RCC, PHEO
P8 (29)	2024	M	51	Rio	c.482G>A	No	HB, PNET, PHEO
P9 (30)	2022	M	35	CHINA	c.269 A > T	Yes	RCC, PHEO
P10 (31)	2023	M	5	Japan	c.482G>A	Yes	PHEO
P11 (32)	2021	M	31	Japan	No	Yes	RCC
P12 (33)	2024	M	21	India	No	Yes	RHB, PHEO, KC
P13 (34)	2022	F	34	CHINA	c.233A>G	Yes	RHB, CHB, KC, PC
P14 (35)	2022	F	25	Japan	c.194C>G	Yes	CHB, RCC, PC
P15 (36)	2016	F	66	Canada	No	Yes	CHB, RHB, RCC, PNET
P16 (37)	2018	F	38	CHINA	c.451A>T	No	CHB, RCC, PNET
P17 (38)	2021	M	32	India	No	Yes	RCC, PNET
P18 (39)	2015	M	42	USA	No	No	PC, KC
P19 (40)	2019	M	29	India	c. 340 + 1G > A	Yes	RCC, PC
P20 (41)	2023	M	39	Indonesia	No	Yes	CHB, PC, KC
P21 (42)	2020	F	17	Tunisia	c.191G>C	No	PHEO
P22 (43)	2014	M	20	CHINA	c.473T>C	Yes	PNET, RCC, HB
P23 (44)	2010	M	28	CHINA	No	Yes	CHB
P24 (45)	2022	M	37	Brazil	No	No	CHB
P25 (46)	2019	M	54	CHINA	c.530-536del	Yes	CHB, RCC, KC
P26 (46)	2019	F	31	CHINA	c.530-536del	Yes	PC, KC
P27 (47)	2021	M	22	CHINA	No	Yes	CHB, PHEO, RCC, PNET
P28 (48)	2024	M	30	France	No	No	CHB, PHEO, PNET, ELST
P29 (49)	2023	M	38	CHINA	No	No	CHB, RCC, PC
P30 (50)	2017	F	38	CHINA	No	Yes	RCC, PC, KC
P31 (51)	2020	F	16	Lithuania	No	No	RCC, CHB
P32 (52)	2024	F	28	Japan	No	Yes	PHEO
P33 (53)	2013	M	41	Turkiye	No	No	CHB
P34 (54)	2022	M	30	Brazil	c.482G>A	Yes	RHB, PHEO, PNET
P35 (55)	2022	M	50	Peru	c.227_229	No	CHB, PHEO, KC, PNET, RCC
P36 (56)	2018	F	51	Japan	No	Yes	CHB, PHEO, PNET
P37 (57)	2015	M	33	CHINA	c.194C>G	No	RCC
P38 (58)	2021	M	40	Japan	No	No	CHB, RCC
P39 (59)	2017	F	39	Japan	No	No	PNET
P40 (60)	2017	F	31	Iran	c.481C>T	No	CHB, PNET, PC, RCC
P41 (61)	2022	M	50	Japan	No	Yes	PHEO, PNET
P42 (62)	2023	M	27	Italy	No	No	RCC, KC
P43 (63)	2022	M	45	Japan	c.194 C>G	Yes	CHB, RCC
P44 (64)	2025	M	39	Russia	c.481C>T	No	CHB
P45 (65)	2019	F	37	India	No	No	CHB, RCC
P46 (66)	2015	M	74	Korea	c.208G>A	Yes	CHB, RHB, RCC
P47 (67)	2016	M	17	CHINA	No	No	CHB, PC, KC
P48 (68)	2020	M	40	CHINA	c.464-1G > C	Yes	CHB, PC, KC
P49 (68)	2020	F	58	CHINA	c.464-2A > G	No	CHB, PC, KC, RCC
P50 (69)	2024	F	25	USA	c.233A>G	No	CHB, PC, KC
P51 (70)	2017	F	40	German	No	No	HB, PNET, PC
P52 (71)	2013	F	27	USA	No	No	PNET, RCC
P53 (72)	2014	M	18	Japan	No mentioned	No	CHB, PNET, PHEO
P54 (73)	2023	F	40	Singao	No	No	RCC, CHB, PC
P55 (74)	2024	F	65	UK	No mentioned	No	CHB, RCC, PHEO, KC
P56 (75)	2015	F	38	Korea	No mentioned	No Mentioned	CHB, RHB, RCC, PC
P57 (75)	2015	M	37	Korea	No mentioned	No Mentioned	RCC
P58 (76)	2023	M	30	India	No	No Mentioned	RHB, CHB

## 4 Conclusion

This case illustrates several important clinical lessons. First, it highlights the importance of recognizing VHL syndrome as a possible underlying cause of pancreatic endocrine insufficiency in young patients with unexplained diabetes. Second, it demonstrates the value of multidisciplinary collaboration among endocrinologists, oncologists, radiologists, and genetic counselors in managing complex VHL cases. Third, it underscores the need for regular long-term surveillance in VHL patients, as new lesions—such as the retinal hemangioblastoma identified during follow-up in this patient—can develop over time.

However, we acknowledge the limitations of a single case report. The associations observed in this patient—including unilateral testicular hypoplasia and osteoporosis—remain speculative and should not be interpreted as established VHL manifestations. Further studies and accumulated case evidence are needed to clarify the mechanisms linking VHL-related pancreatic involvement to endocrine and metabolic complications.

In clinical practice, we recommend that clinicians remain vigilant for pancreatic dysfunction in VHL patients, particularly those with prior pancreatic resections. Early recognition of hyperglycemia may prevent progression to DKA. Continued efforts to understand the pathophysiology of VHL, optimize surveillance protocols, and strengthen genetic counseling are essential to improve long-term outcomes in this patient population.
