# Supplementary material for: Diabetic ketoacidosis associated with pancreatic involvement in a patient with von Hippel-Lindau syndrome: a case report
Source: Front Endocrinol (Lausanne). 2026 Aug 3;17:1880422. doi: 10.3389/fendo.2026.1880422 (PMC13478855; doi:10.3389/fendo.2026.1880422)
Supplement: Supplementary file 1 [file SupplementaryFile1.docx]

Supplementary Figure 1: The green (or dark-colored) regions represent the **preferred conformational regions** (the most stable regions, corresponding to common secondary structures such as α-helices and β-sheets); the light-colored regions are the **allowed conformational regions** (relatively stable); the blank regions are the **disallowed conformational regions** (characterized by excessive steric hindrance and high energy, which rarely occur in the natural state). Most of the scatter points of the wild type fall within the green/light-colored regions, indicating a reasonable conformation. The scatter point distribution of the mutant type differs from that of the wild type, suggesting that the mutation causes the conformations of some residues to deviate from the stable range.

### Supplementary Figure 2: Clinical timeline of the patient's clinical course from 2009 to 2025.
